# Differential gastrointestinal mortality in Native Hawaiian/Pacific Islander and Asian subgroups in the U.S.: a cross-sectional analysis of national mortality surveillance data 2018–2023

**DOI:** 10.1016/j.lana.2026.101372

**Published:** 2026-01-14

**Authors:** Tiange P. Zhang, Jennifer L. Dodge, Norah A. Terrault, Brian P. Lee

**Affiliations:** a Division of Gastrointestinal and Liver Diseases, University of Southern California Keck School of Medicine, Los Angeles, CA, USA; b Department of Population and Public Health Sciences, University of Southern California Keck School of Medicine, Los Angeles, CA, USA

**Keywords:** Native Hawaiians and Pacific islanders, Asians, Health disparities, Data disaggregation, Gastrointestinal diseases

## Abstract

**Background:**

Asians and Native Hawaiians/Pacific Islanders (NHPIs) comprise 8% of the U.S. population with over 40 subgroups. Health data often aggregate these subpopulations, limiting group-specific estimates. In 2018, modifications to death certificates allowed for the disaggregation of NHPIs from Asians. This study examines differences in gastrointestinal-related mortality between NHPI and Asian adults across all major gastrointestinal disease categories.

**Methods:**

We analyzed mortality data for adults aged ≥25 years from the National Center for Health Statistics 2018–2023. Established definitions classified NHPI and Asian individuals based on single or multi-race listings. Gastrointestinal-related deaths were identified using validated ICD-10 codes and categorized into liver diseases, colorectal cancer, upper gastrointestinal cancers, and non-cancer digestive diseases. Age-standardized mortality rates (ASMRs) per 100,000 and rate ratios were calculated.

**Findings:**

Between 2018 and 2023, there were 3322 gastrointestinal-related deaths among NHPI adults (mean age 63.9 years, standard deviation 14.4; 1373 [41.3%] female decedents) and 47,275 among Asian adults (mean age 70.2 years, standard deviation 14.6; 20,892 [44.2%] female decedents). Overall gastrointestinal-related ASMR for NHPIs was 66.8 (95% CI: 64.5–69.2) per 100,000 adults. NHPIs exhibited higher mortality rates than Asians in all gastrointestinal disease categories. NHPIs had 24% higher overall gastrointestinal-related mortality rate than Asians (RR: 1.24; 95% CI: 1.20–1.29), with 33% higher for liver diseases (RR: 1.33; 95% CI: 1.24–1.44) and 63% higher for non-cancer digestive diseases (RR: 1.63; 95% CI: 1.49–1.77).

**Interpretation:**

NHPIs (vs. Asians) have higher gastrointestinal-related mortality rates, with the greatest disparities observed in liver diseases and non-cancer digestive diseases. By disaggregating data across the full spectrum of gastrointestinal diseases, this study provides a clearer understanding of NHPI-specific disparities and highlights critical areas for targeted public health efforts.

**Funding:**

U.S. National Institute of Diabetes and Digestive and Kidney Diseases.

**Research in context**

**Evidence before this study**

We searched PubMed and Google Scholar using the terms “gastrointestinal diseases” OR “digestive system diseases” OR “gastrointestinal neoplasms” OR “liver diseases” OR “liver cirrhosis” OR “liver neoplasms” AND “Native Hawaiian or Pacific Islander” OR “Asian American” AND “mortality” OR “disparities” for U.S. studies published before February 10th, 2025. Previous studies on gastrointestinal health disparities have primarily focused on aggregated data for Asian Americans and Native Hawaiians/Pacific Islanders (AANHPI) as a single group, limiting insight into group-specific disease burdens. While some studies have examined cardiovascular- and cancer-related disparities with disaggregated Asian subgroups, research evaluating mortality across all major gastrointestinal disease categories, including liver diseases and non-cancer digestive diseases, remains sparse. One study using U.S. mortality data disaggregated NHPI and Asian populations, but focused only on gastrointestinal cancers, leaving other major gastrointestinal and liver disease categories unexamined. No prior national studies have comprehensively assessed gastrointestinal-related mortality disparities between NHPI and Asian adults across a full spectrum of gastrointestinal diseases.

**Added value of this study**

This study is the first to systematically compare gastrointestinal-related mortality between NHPI and Asian adults across all major gastrointestinal disease categories, and uses the most up-to-date disaggregated U.S. national mortality data from 2018 to 2023. NHPI adults had significantly higher overall gastrointestinal-related mortality than Asian adults, with disparities most pronounced in liver diseases (33% higher) and non-cancer digestive diseases (63% higher). These findings suggest that previous studies aggregating NHPI and Asian populations may have obscured crucial disparities in a range of gastrointestinal diseases, particularly in liver diseases and non-cancer digestive diseases, which were previously unexamined.

**Implications of all the available evidence**

The identification of disproportionately high gastrointestinal-related mortality rates among NHPIs compared to Asians, particularly in liver diseases and non-cancer digestive diseases, highlights specific areas for further research and targeted public health interventions. These findings also emphasize the importance of continued racial and ethnic data disaggregation in health data to uncover disparities that may be masked by aggregation. Additionally, further research is needed to investigate the underlying drivers of these disparities among NHPI populations, including lifestyle risk factors, socioeconomic factors, healthcare access, and genetic predispositions. These efforts are critical to informing effective policies and public health strategies aimed at reducing gastrointestinal-related mortality disparities in NHPI populations.

## Introduction

The Asian population is the fastest-growing racial group in the U.S., projected to double in the next four decades.^1^ This diverse population comprises over 40 origin groups, but is categorized at times as a monolith in media and healthcare.^2^ Native Hawaiians and Pacific Islanders (NHPI), numbering approximately 1.6 million in 2020, have historically been grouped with Asians under the collective term Asian Americans, Native Hawaiians, and Pacific Islanders (AANHPI).^3^ Descended from the indigenous peoples of Polynesia, Melanesia, and Micronesia, NHPIs may have distinct genetic ancestry, and a unique culture influenced by colonialism, displacement, and cultural assimilation, which may impact health outcomes.^4^^,^^5^

U.S. federal initiatives and medical societies, including in gastroenterology,^6^ have called for the disaggregation of AANHPI data.^7^^,^^8^ Combining all Asian groups together may mask subgroup differences and health disparities.^6^^,^^9^ Emerging evidence from other health domains suggests that NHPIs may face health disparities obscured within the aggregated AANHPI data.^10^, ^11^, ^12^ In gastrointestinal health, studies examining NHPI disparities have focused on gastrointestinal cancers,^13^, ^14^, ^15^ rather than non-cancer digestive diseases and liver diseases among NHPIs. A study using National Center for Health Statistics data from 2018 to 2020 found that aggregated cancer mortality reflected Asian rather than NHPI-specific mortality patterns; however, this study focused on esophageal, gastric, colorectal, liver, and pancreatic cancers.^15^ To date, no national study has evaluated NHPI disparities across the full spectrum of gastrointestinal diseases, leaving the disease categories with the greatest disparities unidentified. Additionally, earlier estimates of gastrointestinal diseases need updates using newly available mortality data through 2023. This study addresses these critical gaps by leveraging the most current disaggregated mortality data to provide a comprehensive national analysis of gastrointestinal disease burden among NHPIs.

Modified death certificate categories in 2018 enabled separating NHPIs from Asians in mortality surveillance across cancer and non-cancer conditions, providing a timely opportunity to characterize NHPI gastrointestinal disease burden nationally. This study conducts the first nationwide comparison of gastrointestinal-related mortality between NHPI and Asian adults across all major gastrointestinal disease categories, including liver diseases, gastrointestinal cancers, and non-cancer digestive diseases, using the most up-to-date mortality data from 2018 to 2023.

## Methods

### Study design

This cross-sectional analysis used national mortality data from the National Center for Health Statistics, accessed via Centers for Disease Control and Prevention (CDC) Wide-ranging Online Data for Epidemiologic Research (WONDER) Multiple Cause of Death database.^16^ These files provide comprehensive death certificate data, including demographics, location, and cause-of-death codes. The analysis used the latest data where NHPIs were disaggregated from Asians, which spanned 2018 to 2023. Deaths attributed to gastrointestinal diseases were identified using ICD-10 codes for the underlying cause of death, encompassing liver diseases (including liver cancer and alcohol-related liver disease), colorectal cancer, upper gastrointestinal cancers (i.e., esophageal, gastric, pancreatic, gallbladder), and non-cancer digestive diseases (i.e., *Clostridium difficile* colitis, ulcers, vascular disorders of the intestine, paralytic ileus and intestinal obstruction, diverticular disease, perforation of intestine, cholecystitis, acute pancreatitis, and gastrointestinal hemorrhage), in accordance with established definitions.^17^, ^18^, ^19^, ^20^ Race and ethnicity information on death certificates were used to stratify mortality data. This study complied with CDC WONDER data use agreement. As the data used were publicly available and deidentified, this study was considered non-human subject research and exempt from the University of Southern California institutional review board. Reporting adhered to Strengthening the Reporting of Observational Studies in Epidemiology (STROBE) guidelines for observational studies.^21^

### Race and ethnicity definitions

Following established definitions,^10^ decedents were categorized as NHPI if their race was recorded as NHPI on death certificates, either as a single race or in combination with other race groups, irrespective of Hispanic or Latino origin. Limiting the definition of the NHPI population to non-Hispanic individuals of a single race risks overlooking the heterogeneity within this group.^7^^,^^10^ Mortality data were also analyzed for other race and ethnicity groups, including for Hispanic or Latino ethnicity and for race groups of White, Black or African American, Asian (not including NHPI), or American Indian or Alaska Native (AIAN). Similar to the definition for NHPI race, we categorized decedents into a race group if the group was specified on the death certificate, regardless of whether it was listed alone or alongside other races and irrespective of Hispanic or Latino origin. Consequently, the race groups are not mutually exclusive, with multi-race individuals included in the age-standardized mortality rates (ASMRs) calculations for every race or ethnicity group indicated on their death certificate.

To assess for potential confounding by individuals who are multi-race, we repeated all analyses separately for decedents of Hispanic or Latino origin, as well as for single-race, non-Hispanic decedents, defined as those with White, Black or African American, Asian, NHPI, or AIAN listed as their only race on the death certificate (Supplemental Table S1).

### Statistical analysis

To calculate gastrointestinal-related age-standardized mortality rates (ASMRs) for each racial and ethnic group, respective death counts (numerator) were extracted from the CDC WONDER Multiple Cause of Death database^16^ and aggregated across the six-year period from 2018 to 2023. The respective population counts (denominator) were obtained from the U.S. Census Bureau's population estimate files^22^ and aggregated across the six-year period from 2018 to 2023. Population estimate files were sourced from the June 2021 release for 2018 to 2019, the June 2023 release for 2020 to 2022, and the June 2024 release for 2023. Death counts and population counts extracted for each racial category included individuals identified alone or in combination with other races.^10^ ASMR calculations standardized mortality rates to the 2000 US standard population using the direct method in 10-year age groups (25–34, 35–44, 45–54, 55–64, 65–74, 75–84, and ≥85). ASMRs were expressed per 100,000 individuals. For each ASMR, 95% confidence intervals (CIs) were calculated. Rate ratios (RRs), along with their 95% CIs, were computed to facilitate comparisons of ASMRs between NHPI and Asian populations, as well as between NHPI and other racial or ethnic groups. For all RR calculations, the ASMR for NHPI served as the numerator and the ASMR for the non-NHPI group served as the denominator or reference group, so that RR > 1 indicates higher mortality in the NHPI group. Individual-level age data were not available due to CDC WONDER suppression of cells with fewer than 10 events; therefore, mean age and standard deviation were calculated using the midpoint method for grouped data.

In an exploratory analysis focused on non-cancer digestive diseases, we grouped etiologies closely related to gastrointestinal bleeding - hematemesis/melena/gastrointestinal hemorrhage, ulcer diseases, and vascular disorders of the intestine - into a “bleeding non-cancer digestive diseases” category, with remaining non-cancer digestive diseases classified as non-bleeding (Supplemental Table S3). Analyses were restricted to adults aged ≥35 years due to suppression of NHPI age-specific death counts for some etiology groups among ages ≥25 years in CDC WONDER, which precluded ASMR calculation.

In a sensitivity analysis, we calculated year-specific ASMRs for gastrointestinal diseases among NHPI and Asian adults aged ≥25 years for 2019 to 2023 (Supplemental Table S4). Because certain NHPI age-specific death counts for 2018 were suppressed in CDC WONDER due to low counts, NHPI ASMR could not be calculated for that year. For each calendar year, we calculated ASMRs using the same direct age-standardization approach and derived rate ratios comparing NHPI with Asian adults to assess whether disparities were consistent before and after the COVID-19 period.

Statistical analyses were performed using SAS (version 9.4, SAS Institute Inc, Cary, North Carolina) and Stata (version 18.5, StataCorp LLC, College Station, Texas) software.

### Role of Funding Source

The funders had no role in the design and conduct of the study; collection, management, analysis, and interpretation of the data; preparation, review, or approval of the manuscript; and decision to submit the manuscript for publication.

## Results

From 2018 to 2023, there were 3322 gastrointestinal-related deaths among NHPI adults and 47,275 deaths among Asian adults (Table 1). A higher proportion of NHPI deaths occurred before the age of 65 years compared to Asian deaths (1660 [50.0%] vs. 15,341 [32.5%]). A higher percentage of NHPI decedents were male (1949 [58.7%] vs. 26,383 [55.8%]) and multi-race (1392 [41.9%] vs. 2838 [6.0%]) compared to Asian decedents. Geographically, 2756 (83.0%) of NHPI deaths occurred in nine states, with the largest concentrations in Hawai'i (1266 [38.1%]) and California (697 [21.0%]). In contrast, 29,397 (62.2%) of Asian gastrointestinal-related deaths occurred in the same nine states.

**Table 1:** Characteristics of adults ≥25 years in the U.S. with gastrointestinal diseases as the underlying cause of death by NHPI or Asian race group, 2018 to 2023.

	Gastrointestinal diseases			
	NHPI, %		Asian, %	
Deaths	**3322**		**47,275**	
Sex				
Male	1949	58.7	26,383	55.8
Female	1373	41.3	20,892	44.2
Age Group				
25–64	1660	50.0	15,341	32.5
25–34	89	2.7	599	1.3
35–44	229	6.9	1923	4.1
45–54	520	15.7	4507	9.5
55–64	822	24.7	8312	17.6
65–74	872	26.2	11,935	25.2
75–84	546	16.4	11,507	24.3
85+	244	7.3	8492	18.0
Race/Ethnicity				
Single race	1930	58.1	44,437	94.0
Non-Hispanic	1760	53.0	43,902	92.9
Hispanic	170	5.1	535	1.1
Multi-race	1392	41.9	2838	6.0
Non-Hispanic	1273	38.3	2524	5.3
Hispanic	119	3.6	314	0.7
Home State				
9 states combined	2756	83.0	29,397	62.2
Hawai'i	1266	38.1	3507	7.4
California	697	21.0	17,473	37.0
Washington	221	6.7	1857	3.9
Nevada	113	3.4	891	1.9
Texas	110	3.3	2725	5.8
Utah	118	3.6	169	0.4
Florida	69	2.1	1578	3.3
Oregon	89	2.7	491	1.0
Arizona	73	2.2	706	1.5

NHPI = Native Hawaiian and Pacific Islander.

### Gastrointestinal-related mortality

**Fig. 1:**
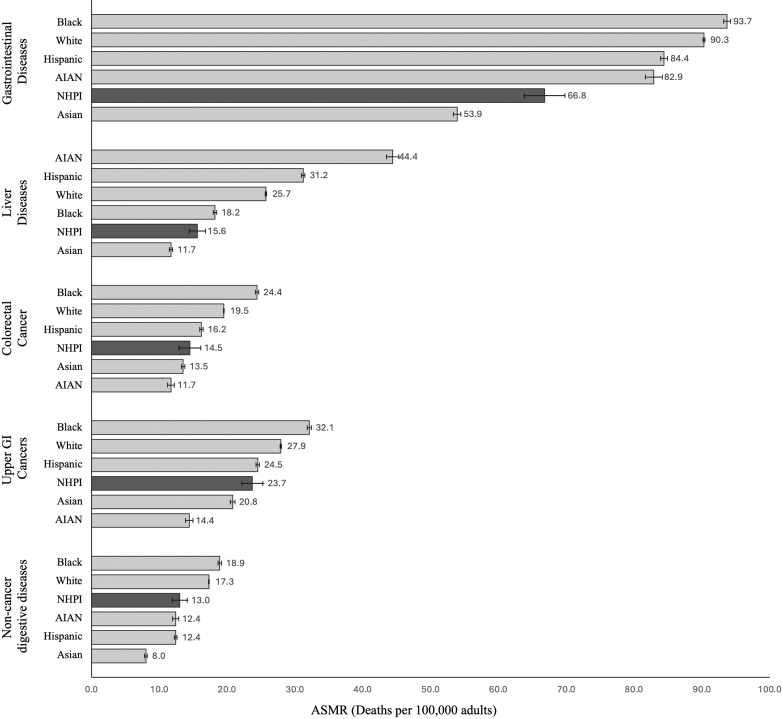
Gastrointestinal-related age-standardized mortality rates (ASMRs) and 95% CIs among adults ≥25 years in the U.S. from 2018 to 2023. Validated international classification of diseases 10th revision (ICD-10) codes for the underlying cause of death were used to categorize deaths as attributable to gastrointestinal diseases, comprising of liver diseases (including liver cancer), colorectal cancer, upper gastrointestinal cancers (i.e., esophageal, gastric, pancreatic, gallbladder), and non-cancer digestive diseases (e.g., gastrointestinal hemorrhage, acute pancreatitis, etc.). AIAN = American Indian/Alaska Native; NHPI = Native Hawaiian and Pacific Islander.

Between 2018 and 2023, the age-standardized mortality rate (ASMR) for gastrointestinal diseases among NHPIs was 66.8 deaths per 100,000 adults (95% CI: 64.5–69.2) (Fig. 1, Supplemental Table S2). When compared to other racial and ethnic groups, the ASMR for NHPIs exceeded that of Asians (53.9 per 100,000; 95% CI: 53.4–54.4) and was lower than AIANs (82.9 per 100,000; 95% CI: 81.8–84.1), Hispanics (84.4 per 100,000; 95% CI: 83.9–84.8), Blacks (93.7 per 100,000; 95% CI: 93.2–94.1), and Whites (90.3 per 100,000; 95% CI: 90.2–90.5). The ASMR for gastrointestinal diseases among NHPI adults was 24% higher (RR: 1.24; 95% CI: 1.20–1.29) compared to Asian adults.

### Liver diseases-related mortality

The ASMR for liver diseases among NHPIs was 15.6 deaths per 100,000 adults (95% CI: 14.5–16.7) (Supplemental Table S2). Compared to other racial and ethnic groups, the NHPI ASMR exceeded that of Asians (11.7 per 100,000; 95% CI: 11.4–11.9) but was lower than AIANs (44.4 per 100,000; 95% CI: 43.6–45.2), Hispanics (31.2 per 100,000; 95% CI: 31.0–31.5), Blacks (18.2 per 100,000; 95% CI: 18.0–18.4), and Whites (25.7 per 100,000; 95% CI: 25.6–25.8). The ASMR for liver diseases among NHPI adults was 1.33 times higher (RR: 1.33; 95% CI: 1.24–1.44) than that of Asian adults. The ASMR for alcohol-associated liver disease was 4.9 deaths per 100,000 adults (95% CI: 4.3–5.5) for NHPIs compared to 2.9 deaths per 100,000 adults (95% CI: 2.8–3.0) among Asians (RR: 1.69, 95% CI: 1.49–1.92) (Table 2). For non-alcohol associated liver diseases, the ASMR was 10.7 deaths per 100,000 adults (95% CI: 9.8–11.6) for NHPIs and 8.8 deaths per 100,000 adults (95% CI: 8.6–9.0) for Asians (RR: 1.22, 95% CI: 1.11–1.33).

**Table 2:** Liver disease-related mortality by alcohol-associated and non-alcohol associated etiologies among adults aged ≥25 years by established race group, 2018 to 2023.

	2018 to 2023					
	Deaths^a^	Population	ASMR (per 100,000)	(95% CI)	RR^b^	(95% CI)
Alcohol associated liver disease						
NHPI	272 (33.0%)	5,854,506	4.9	(4.3–5.5)	–	
Asian	2682 (25.6%)	95,148,011	2.9	(2.8–3.0)	1.69	(1.49–1.92)
Non-alcohol associated liver diseases						
NHPI	552 (67.0%)	5,854,506	10.7	(9.8–11.6)	–	
Asian	7815 (74.4%)	95,148,011	8.8	(8.6–9.0)	1.22	(1.11–1.33)

ASMR, age-standardized mortality rate. NHPI, Native Hawaiian and Pacific Islander.

a ICD-10 codes for alcohol-associated liver disease included: K70.0 (Alcoholic fatty liver); K70.1 (Alcoholic hepatitis); K70.2 (Alcoholic fibrosis and sclerosis of liver); K70.3 (Alcoholic cirrhosis of liver); K70.4 (Alcoholic hepatic failure); K70.9 (Alcoholic liver disease, unspecified). Percentages are within race group and represent the proportion of liver disease-related deaths attributable to each etiology.

b Rate ratios (RR) compared NHPI to each listed non-NHPI group (reference), such that RR > 1 indicates higher mortality in NHPIs.

### Colorectal cancer-related mortality

The ASMR for colorectal cancer among NHPIs was 14.5 deaths per 100,000 adults (95% CI: 13.4–15.6) (Supplemental Table S2). Compared to other racial and ethnic groups, the NHPI ASMR was slightly higher than that of Asians (13.5 per 100,000; 95% CI: 13.2–13.7) and higher than that of AIANs (11.7 per 100,000; 95% CI: 11.3–12.2) but was lower than Hispanics (16.2 per 100,000; 95% CI: 16.0–16.4), Blacks (24.4 per 100,000; 95% CI: 24.2–24.7), and Whites (19.5 per 100,000; 95% CI: 19.4–19.5). When compared using RR, the ASMR for colorectal cancer in NHPI adults was similar to or slightly higher than that of Asian adults (RR: 1.07; 95% CI: 0.99–1.16).

### Upper gastrointestinal cancer-related mortality

The ASMR for upper gastrointestinal cancer among NHPIs was 23.7 deaths per 100,000 adults (95% CI: 22.3–25.2) (Supplemental Table S2). Compared to other racial and ethnic groups, the NHPI ASMR exceeded that of Asians (20.8 per 100,000; 95% CI: 20.5–21.1) and AIANs (14.4 per 100,000; 95% CI: 13.9–14.9) but lower than Hispanics (24.5 per 100,000; 95% CI: 24.3–24.8), Blacks (32.1 per 100,000; 95% CI: 31.8–32.4), and Whites (27.9 per 100,000; 95% CI: 27.8–28.0). The ASMR for upper gastrointestinal cancer among NHPI adults was 1.14 times higher (RR: 1.14; 95% CI: 1.07–1.21) than that of Asian adults.

### Non-cancer digestive diseases-related mortality

The ASMR for non-cancer digestive diseases among NHPIs was 13.0 deaths per 100,000 adults (95% CI: 11.9–14.0) (Supplemental Table S2). Compared to other racial and ethnic groups, the NHPI ASMR exceeded that of Asians (8.0 per 100,000; 95% CI: 7.8–8.2), was similar to that of Hispanics (12.4 per 100,000; 95% CI: 12.2–12.6) and AIANs (12.4 per 100,000; 95% CI: 11.9–12.8), and was lower than that of Blacks (18.9 per 100,000; 95% CI: 18.7–19.1) and Whites (17.3 per 100,000; 95% CI: 17.3–17.4). The ASMR for non-cancer digestive diseases among NHPI adults was 1.63 times higher (RR: 1.63; 95% CI: 1.49–1.77) than that of Asian adults. In an exploratory analysis among adults aged ≥35 years (Supplemental Table S3), NHPIs had higher ASMR for non-cancer digestive diseases overall compared with Asians. The disparity was more pronounced for bleeding non-cancer digestive diseases (RR: 1.73; 95% CI: 1.55–1.93), where mortality in NHPIs was higher than that of Asians, and remained elevated for non-bleeding non-cancer digestive diseases as well (RR: 1.47; 95% CI: 1.27–1.69).

### Gastrointestinal-related mortality among single-race adults

Patterns of findings remained unchanged when analyses were repeated separately for decedents of Hispanic or Latino origin, as well as for non-Hispanic, single-race decedents, defined as those with White, Black or African American, Asian, NHPI, or AIAN listed as their only race on the death certificate (Supplemental Table S1). ASMRs for gastrointestinal diseases were consistently higher among non-Hispanic, single-race NHPI adults compared to all NHPI adults in the primary analysis (i.e., those listed as single- or multi-race regardless of Hispanic ethnicity). Rate ratios comparing ASMRs of NHPIs and Asians were also consistently higher for non-Hispanic, single-race group.

### Sensitivity analysis

In our sensitivity analysis by calendar year (Supplemental Table S4), NHPI adults had higher gastrointestinal disease ASMRs than Asian adults in every year from 2019 to 2023. During these years before and after the COVID-19 pandemic, year-specific rate ratios comparing NHPIs with Asians were largely stable over time.

## Discussion

This is the first nationwide study to comprehensively evaluate mortality disparities between NHPI and Asian adults across all major gastrointestinal disease categories, including not only gastrointestinal cancers but also liver diseases and non-cancer digestive diseases. By disaggregating mortality data across all major disease categories, we identified where NHPI disparities may be most pronounced, revealing that disparities in liver diseases and non-cancer digestive diseases are particularly stark. While the overall gastrointestinal-related mortality rate among NHPIs was 24% higher than that of Asians, the disparity was even greater for these specific categories, with mortality rates 33% higher for liver diseases and 63% higher for non-cancer digestive diseases. Additionally, half of NHPI gastrointestinal-related deaths occurred before age 65, emphasizing the need for earlier detection and intervention strategies. These findings not only reaffirm the importance of disaggregating NHPI and Asian health data but also pinpoint specific areas where disparities are most severe, providing a critical foundation for future NHPI research and targeted public health strategies.

Our finding revealed that the rate ratio comparing NHPIs to Asians for non-cancer digestive disease mortality was the highest among all gastrointestinal disease categories. Unlike previous studies that primarily focused on gastrointestinal cancer disparities,^13^, ^14^, ^15^ our findings showed that NHPIs may face an even greater mortality disparity in non-cancer digestive diseases, highlighting a previously unrecognized gap. Consistent with prior research using National Center for Health Statistics mortality data, gastrointestinal bleeding accounted for the largest proportion of deaths within the non-cancer digestive diseases category.^23^ In our exploratory analysis, NHPIs also had a mortality rate from bleeding non-cancer digestive diseases that was about 1.73 times that of Asians. Racial disparities in gastrointestinal bleeding mortality may be driven in part by differences in access to emergency gastrointestinal services, including timely endoscopy.^24^ Given that NHPI populations may be more distant from facilities offering urgent gastrointestinal interventions,^25^, ^26^, ^27^ delays in care could contribute to their disproportionately high mortality from acute digestive conditions such as gastrointestinal hemorrhage and peptic ulcer disease. Further research is needed to assess the role of emergency gastrointestinal care access in driving disparities in non-cancer digestive disease outcomes between NHPI and Asian populations.

Liver-related mortality is significantly higher in NHPI compared to Asian adults, particularly for alcohol-associated liver disease. While previous studies have highlighted higher hepatocellular carcinoma rates in NHPI,^15^ our findings revealed a broader disparity in total liver-related mortality including cirrhosis. Given that over 90% of hepatocellular carcinoma in the U.S. occurs in the setting of cirrhosis or advanced fibrosis,^28^ assessing total liver-related mortality provides a more comprehensive picture of NHPI's chronic liver disease burden, with alcohol-associated liver disease emerging as a key driver. One possible explanation for this disparity may be the interaction between alcohol use and metabolic dysfunction, which may disproportionately impact NHPIs due to a combination of genetic and behavioral factors. For instance, East Asians have high prevalence of aldehyde dehydrogenase 2 deficiency, which may modulate alcohol consumption and associated liver damage, while it remains unclear whether NHPIs share this genetic trait.^29^^,^^30^ Additionally, studies show NHPIs may exhibit a distinct liver fat storage pattern, with higher liver fat accumulation relative to total body fat and greater visceral adiposity compared to Asians,^31^ which may further increase NHPI population's risk of liver disease progression even in the absence of alcohol use. When compounded with NHPI's higher alcohol consumption rates,^32^ these vulnerabilities may accelerate liver disease progression. The synergistic effect of alcohol and metabolic syndrome also accelerates liver disease progression, as alcohol exacerbates obesity, hypertension, and insulin resistance, leading to faster fibrosis and more severe cirrhosis than either factor alone.^33^^,^^34^ This effect is particularly concerning in NHPIs, who already have a higher burden of metabolic syndrome compared to Asians.^32^ These findings highlight the need for targeted interventions to address the combined impact of alcohol use and metabolic dysfunction in NHPI populations.

While disparities were most pronounced in non-cancer digestive diseases and liver diseases, NHPI adults had higher mortality rates across all major gastrointestinal disease categories compared to Asians. Although our analysis focused on mortality, prior SEER-based study has characterized gastrointestinal cancer incidence among Asian subgroups and NHPIs.^35^ That study reported higher colorectal cancer and esophageal adenocarcinoma incidence in NHPI men than in most Asian subgroups, whereas incidence of most other cancers, including esophageal squamous cell carcinoma and noncardia and cardia gastric cancers, was similar or lower in NHPIs. Taken together, these findings suggest that excess NHPI mortality may reflect more than differences in incidence but also downstream factors such as stage at diagnosis, access to timely treatment, and quality of care. A 2014 national survey found that NHPI individuals were more likely to rely on public health coverage and experience delays in medical care compared to Asians.^32^ Among NHPIs, cultural preferences for non-Western practitioners and a shortage of ethnically concordant physicians may further contribute to medical mistrust.^36^^,^^37^ Additionally, NHPI individuals in remote regions may face limited access to subspecialty care, including gastroenterologists and liver transplant centers.^25^, ^26^, ^27^ These barriers may restrict access to preventive services, timely diagnosis, and high-quality care. Addressing these structural causes requires action at provider, community, system, and policy levels. One approach is to apply disaggregation across the entire data life cycle from data collection to dissemination.^14^ Broader policy changes including increased funding for NHPI research are also needed.^7^ Community-based interventions tailored to NHPI populations could also help bridge gaps in care by leveraging culturally relevant approaches.^38^, ^39^, ^40^

This study has several strengths, including the use of a fully encompassing national dataset and the disaggregation of AANHPI population. The comprehensive data enabled the identification of specific disparities across categories of gastrointestinal conditions, such as elevated mortality rates in liver diseases and non-cancer digestive diseases among NHPIs compared to Asians.

This study had limitations. First, race and ethnicity data recorded on death certificates may be subject to misclassification bias, as classification often relies on observation rather than self-report.^41^ This may lead to under-ascertainment of deaths among NHPI individuals. Second, misclassification of the underlying cause of death has also been reported; however, the degree to which the underlying cause of death is inconsistently classified for NHPI decedents relative to other racial or ethnic groups is not anticipated to be different. Third, while this analysis benefitted from recent mortality data following the 2018 disaggregation of NHPI and Asian populations, the generalizability of these findings to earlier periods before 2018 is uncertain. Fourth, WONDER lacks individual-level data, and the contribution of demographic, biologic, and social determinants of health factors with regards to our results could not be rigorously addressed. Finally, NHPIs represent a relatively small population, which limited more granular assessments of gastrointestinal etiologies. Among liver-related etiologies, additional stratification by metabolic dysfunction-associated steatotic liver disease (MASLD) was limited by suppression of several NHPI age-specific death counts in CDC WONDER, which precluded ASMR estimation. Additionally, gastrointestinal-related death counts for younger age groups were frequently suppressed in CDC WONDER as well due to low counts, which precluded ASMR estimation for young adults and adolescents. Because NHPIs represent a relatively small population, their ASMRs had somewhat wider confidence intervals than those of other groups. However, all estimates were derived from cells with >20 deaths, in line with CDC WONDER guidance for rate stability. Future work should prioritize ongoing, disaggregated data collection for NHPI populations to allow more precise estimates and additional assessment of subgroup differences over time. Additionally, NHPI itself is not a monolithic group, encompassing diverse cultural backgrounds, and future analyses should aim to disaggregate data within NHPI subpopulations to better understand potential differences in disease burden and mechanisms of disparity. Overall, our results identify and describe important disparities, which should prompt further investigation into the underlying etiologies driving these disparities and the identification of specific targets for intervention to improve public health outcomes.

In conclusion, this national study provides critical insights into previously unexplored disparities in gastrointestinal-related mortality among NHPI adults compared to Asian adults, revealing significantly higher mortality not only overall but particularly in liver diseases and non-cancer digestive diseases among NHPIs. By identifying disparities in these specific disease categories, our findings highlight key directions for future NHPI research. These results also underscore the importance of continued disaggregation of NHPI populations in public health research and surveillance to uncover disparities that may have been masked by prior aggregation. Such efforts are essential for advancing our understanding of NHPI health burdens while informing more effective resource allocation and personalized intervention strategies for gastrointestinal health.

## Contributors

Conceptualization: T.P.Z., B.P.L.; Methodology: T.P.Z., J.L.D., N.A.T., B.P.L.; Formal Analysis: T.P.Z.; Data Curation: T.P.Z.; Writing-original draft: T.P.Z.; Writing-review and editing: T.P.Z., J.L.D., N.A.T., B.P.L.; Supervision: B.P.L., T.P.Z., and B.P.L. have directly accessed and verified the underlying data reported in the manuscript. All authors approved the final manuscript; B.P.L. made the final decision to submit for publication.

## Data sharing statement

Public data were used for this study. Study protocol, statistical analysis plan, and analytic code will be available upon publication with no end date. Access can be requested from the corresponding author.

## Declaration of interests

We declare no competing interests.
