# Supplementary material for: Differential gastrointestinal mortality in Native Hawaiian/Pacific Islander and Asian subgroups in the U.S.: a cross-sectional analysis of national mortality surveillance data 2018–2023
Source: Lancet Reg Health Am. 2026 Jan 14;55:101372. doi: 10.1016/j.lana.2026.101372 (PMC12829122; doi:10.1016/j.lana.2026.101372)
Supplement: Supplementary Tables [file mmc1.pdf]

Differential Gastrointestinal Mortality in Native Hawaiian/Pacific Islander and Asian Subgroups in the U.S.: A Cross-Sectional Analysis of National Mortality Surveillance Data 2018-2023

Supplementary Material: Table of Contents

1. **Supplemental Table 1.** Gastrointestinal-related mortality rates among adults by single-race groups, 2018 to 2023
2. **Supplemental Table 2.** Gastrointestinal-related mortality rates among adults by established race groups, 2018 to 2023
3. **Supplemental Table 3.** Non-cancer digestive diseases-related mortality by bleeding and non-bleeding etiologies among NHPI and Asian adults aged ≥ 35 years by established race group, 2018 to 2023
4. **Supplemental Table 4.** Mortality rates of gastrointestinal diseases among NHPI and Asian adults aged ≥ 25 years by established race groups and by calendar year, 2019 to 2023

Supplemental Table 1. Gastrointestinal-related mortality rates among adults by single-race groups, 2018 to 2023

	2018 to 2023					
	**Deaths[a]**	**Population**	**ASMR (per 100,000)**	**(95% CI)**	**RR[b]**	**(95% CI)**
**Gastrointestinal diseases**						
NHPI	1,760	2,417,253	79.6	(75.7-83.4)	—	
Black	160,864	163,850,503	98.4	(98.0-98.9)	0.81	(0.77-0.85)
White	1,041,697	867,128,466	90.9	(90.7-91.1)	0.88	(0.83-0.92)
Hispanic	146,738	217,503,176	84.4	(83.9-84.8)	0.94	(0.90-0.99)
Asian	43,902	83,828,956	54.5	(54.0-55.1)	1.46	(1.39-1.53)
AIAN	15,581	9,522,861	162.6	(159.9-165.2)	0.49	(0.47-0.52)
**Liver diseases**						
NHPI	436	2,417,253	18.9	(17.0-20.7)	—	
Black	33,680	163,850,503	19.1	(18.9-19.3)	0.99	(0.90-1.09)
White	262,862	867,128,466	24.8	(24.7-24.9)	0.76	(0.69-0.84)
Hispanic	59,206	217,503,176	31.2	(31.0-31.5)	0.61	(0.55-0.67)
Asian	9,496	83,828,956	11.6	(11.4-11.8)	1.63	(1.48-1.80)
AIAN	9,210	9,522,861	96.4	(94.3-98.4)	0.20	(0.18-0.22)
**Colorectal cancer**						
NHPI	408	2,417,253	18	(16.2-19.8)	—	
Black	41,543	163,850,503	25.8	(25.5-26.0)	0.70	(0.63-0.77)
White	228,920	867,128,466	19.9	(19.8-20.0)	0.90	(0.82-1.00)
Hispanic	27,493	217,503,176	16.2	(16.0-16.4)	1.11	(1.00-1.23)
Asian	11,133	83,828,956	13.7	(13.5-14.0)	1.31	(1.19-1.45)
AIAN	1,914	9,522,861	19.8	(18.9-20.7)	0.91	(0.81-1.01)
**Upper gastrointestinal cancers**						
NHPI	604	2,417,253	27.5	(25.2-29.8)	—	
Black	55,179	163,850,503	33.7	(33.5-34.0)	0.82	(0.75-0.89)
White	338,139	867,128,466	28.2	(28.1-28.3)	0.98	(0.90-1.06)
Hispanic	40,896	217,503,176	24.5	(24.3-24.8)	1.12	(1.03-1.22)
Asian	17,060	83,828,956	21.2	(20.9-21.5)	1.30	(1.19-1.41)
AIAN	2,404	9,522,861	24.2	(23.2-25.2)	1.14	(1.04-1.25)
**Non-cancer digestive diseases**						
NHPI	312	2,417,253	15.2	(13.5-16.9)	—	
Black	30,462	163,850,503	19.9	(19.7-20.1)	0.76	(0.68-0.85)
White	211,776	867,128,466	18	(17.9-18.1)	0.84	(0.75-0.94)
Hispanic	19,143	217,503,176	12.4	(12.2-12.6)	1.23	(1.09-1.37)
Asian	6,213	83,828,956	8	(7.8-8.2)	1.90	(1.69-2.13)
AIAN	2,053	9,522,861	22.2	(21.2-23.2)	0.68	(0.61-0.77)

[a]ICD-10 codes for the underlying cause of death were used to categorize deaths as attributable to gastrointestinal diseases, comprising of liver diseases (including liver cancer), colorectal cancer, upper gastrointestinal cancers (i.e., esophageal, gastric, pancreatic, gallbladder), and non-cancer digestive diseases (e.g., gastrointestinal hemorrhage, acute pancreatitis, etc.).

[b]Rate ratios (RR) compared NHPI to each listed non-NHPI group (reference), such that RR > 1 indicates higher mortality in NHPIs.

AIAN, American Indian and Alaska Native. ASMR, age-standardized mortality rate. NHPI, Native Hawaiian and Pacific Islander.

Supplemental Table 2. Gastrointestinal-related mortality rates among adults by established race groups, 2018 to 2023

	2018 to 2023					
	Deaths[a]	Population	ASMR (per 100,000)	(95% CI)	RR[b]	(95% CI)
**Gastrointestinal diseases**						
NHPI	3,322	5,854,506	66.8	(64.5-69.2)	—	
Black	164,749	183,161,379	93.7	(93.2-94.1)	0.71	(0.69-0.74)
White	1,193,178	1,081,624,608	90.3	(90.2-90.5)	0.74	(0.71-0.77)
Hispanic	146,738	217,503,176	84.4	(83.9-84.8)	0.79	(0.76-0.82)
Asian	47,275	95,148,011	53.9	(53.4-54.4)	1.24	(1.20-1.29)
AIAN	20,555	25,733,474	82.9	(81.8-84.1)	0.81	(0.78-0.84)
**Liver diseases**						
NHPI	824	5,854,506	15.6	(14.5-16.7)	—	
Black	34,900	183,161,379	18.2	(18.0-18.4)	0.86	(0.80-0.92)
White	323,730	1,081,624,608	25.7	(25.6-25.8)	0.61	(0.57-0.65)
Hispanic	59,206	217,503,176	31.2	(31.0-31.5)	0.50	(0.47-0.54)
Asian	10,497	95,148,011	11.7	(11.4-11.9)	1.33	(1.24-1.44)
AIAN	11,451	25,733,474	44.4	(43.6-45.2)	0.35	(0.33-0.38)
**Colorectal cancer**						
NHPI	743	5,854,506	14.5	(13.4-15.6)	—	
Black	42,458	183,161,379	24.4	(24.2-24.7)	0.59	(0.55-0.64)
White	257,373	1,081,624,608	19.5	(19.4-19.5)	0.74	(0.69-0.80)
Hispanic	27,493	217,503,176	16.2	(16.0-16.4)	0.90	(0.83-0.97)
Asian	11,914	95,148,011	13.5	(13.2-13.7)	1.07	(0.99-1.16)
AIAN	2,785	25,733,474	11.7	(11.3-12.2)	1.24	(1.14-1.35)
**Upper gastrointestinal cancers**						
NHPI	1,163	5,854,506	23.7	(22.3-25.2)	—	
Black	56,305	183,161,379	32.1	(31.8-32.4)	0.74	(0.69-0.79)
White	380,177	1,081,624,608	27.9	(27.8-28.0)	0.85	(0.80-0.90)
Hispanic	40,896	217,503,176	24.5	(24.3-24.8)	0.97	(0.91-1.03)
Asian	18,151	95,148,011	20.8	(20.5-21.1)	1.14	(1.07-1.21)
AIAN	3,499	25,733,474	14.4	(13.9-14.9)	1.65	(1.53-1.77)
**Non-cancer digestive diseases**						
NHPI	592	5,854,506	13.0	(11.9-14.0)	—	
Black	31,086	183,161,379	18.9	(18.7-19.1)	0.69	(0.63-0.75)
White	231,898	1,081,624,608	17.3	(17.3-17.4)	0.75	(0.69-0.81)
Hispanic	19,143	217,503,176	12.4	(12.2-12.6)	1.05	(0.97-1.14)
Asian	6,713	95,148,011	8.0	(7.8-8.2)	1.63	(1.49-1.77)
AIAN	2,820	25,733,474	12.4	(11.9-12.8)	1.05	(0.96-1.15)

[a]ICD-10 codes for the underlying cause of death were used to categorize deaths as attributable to gastrointestinal diseases, comprising of liver diseases (including liver cancer), colorectal cancer, upper gastrointestinal cancers (i.e., esophageal, gastric, pancreatic, gallbladder), and non-cancer digestive diseases (e.g., gastrointestinal hemorrhage, acute pancreatitis, etc.).
[b]Rate ratios (RR) compared NHPI to each listed non-NHPI group (reference), such that RR > 1 indicates higher mortality in NHPIs.
AIAN, American Indian and Alaska Native. ASMR, age-standardized mortality rate. NHPI, Native Hawaiian and Pacific Islander.

Supplemental Table 3. Non-cancer digestive diseases-related mortality by bleeding and non-bleeding etiologies among NHPI and Asian adults aged ≥ 35 years by established race group, 2018 to 2023

	2018 to 2023					
	**Deaths[a]**	**Population**	**ASMR (per 100,000)**	**(95% CI)**	**RR[b]**	**(95% CI)**
**Non-cancer digestive diseases**						
NHPI	575	4,242,386	16.1	(14.8-17.5)	—	
Asian	6,635	72,148,341	10.0	(9.8-10.3)	1.61	(1.48-1.76)
**Bleeding non-cancer digestive diseases**						
NHPI	344	4,242,386	9.5	(8.5-10.5)	—	
Asian	3,639	72,148,341	5.5	(5.3-5.7)	1.73	(1.55-1.93)
**Non-bleeding non-cancer digestive diseases**						
NHPI	231	4,242,386	6.6	(5.7-7.5)	—	
Asian	2,996	72,148,341	4.5	(4.4-4.7)	1.47	(1.27-1.69)

[a]ICD-10 codes for bleeding non-cancer digestive diseases included: Hematemesis/melena/gastrointestinal hemorrhage (K92.0-92.2), Gastric/duodenal/peptic ulcers (K25-28), Vascular disorders of intestine (K55); for non-bleeding non-cancer digestive diseases included: Enterocolitis due to Clostridium difficile (A04.7), Paralytic ileus and intestinal obstruction without hernia (K56), Diverticular disease of intestine (K57); Perforation of intestine, nontraumatic (K63.1); Cholecystitis (K81); Acute pancreatitis (K85). Analyses were restricted to adults aged ≥35 years due to suppression of NHPI age-specific death counts for some etiology groups among ages ≥25 years in CDC WONDER, which precluded ASMR calculation.

[b]Rate ratios (RR) compared NHPI to each listed non-NHPI group (reference), such that RR > 1 indicates higher mortality in NHPIs.

ASMR, age-standardized mortality rate. NHPI, Native Hawaiian and Pacific Islander.

Supplemental Table 4. Mortality rates of gastrointestinal diseases among NHPI and Asian adults aged ≥ 25 years by established race groups and by calendar year, 2019 to 2023

	Gastrointestinal diseases					
	Deaths[a]	Population	ASMR (per 100,000)	(95% CI)	RR[b]	(95% CI)
**2019**						
NHPI	521	932,435	67.9	(61.8-74.0)	—	
Asian	7,335	15,241,518	53.5	(52.2-54.7)	1.27	(1.16-1.39)
**2020**						
NHPI	554	973,210	69.0	(63.1-75.0)	—	
Asian	7,753	15,687,749	54.3	(53.1-55.6)	1.27	(1.16-1.39)
**2021**						
NHPI	592	993,993	69.0	(63.3-74.7)	—	
Asian	8,340	16,001,348	56.1	(54.9-57.3)	1.23	(1.13-1.34)
**2022**						
NHPI	563	1,016,866	62.3	(57.1-67.6)	—	
Asian	8,294	16,449,700	53.2	(52.1-54.4)	1.17	(1.07-1.28)
**2023**						
NHPI	608	1,029,750	67.0	(61.6-72.5)	—	
Asian	8,488	16,897,711	52.6	(51.5-53.7)	1.27	(1.17-1.39)

[a]ICD-10 codes for the underlying cause of death were used to categorize deaths as attributable to gastrointestinal diseases, comprising of liver diseases (including liver cancer), colorectal cancer, upper gastrointestinal cancers (i.e., esophageal, gastric, pancreatic, gallbladder), and non-cancer digestive diseases (e.g., gastrointestinal hemorrhage, acute pancreatitis, etc.). 2018 was excluded due to suppression of certain NHPI age-specific death counts in CDC WONDER, which precluded ASMR calculation.

[b]Rate ratios (RR) compared NHPI to each listed non-NHPI group (reference), such that RR > 1 indicates higher mortality in NHPIs.

ASMR, age-standardized mortality rate. NHPI, Native Hawaiian and Pacific Islander.
